# Diagnostic challenges of an incidental finding: case report of definitely-congenital glioblastoma multiforme in a very preterm infant

**DOI:** 10.1186/s13052-021-01185-3

**Published:** 2021-12-14

**Authors:** Silvia Martini, Vittoria Paoletti, Monica Maffei, Mino Zucchelli, Chiara Locatelli, Maximilian Fischer, Viscardo Paolo Fabbri, Maria Pia Foschini, Giovanni Tallini, Luigi Corvaglia

**Affiliations:** 1 Neonatal Intensive Care Unit, IRCCS Policlinico di S. Orsola, S. Orsola-Malpighi Hospital, Via Massarenti 11, 40138 Bologna, Italy; 2 Department of Medical and Surgical Sciences (DIMEC), University of Bologna, Bologna, Italy; 3 IRCCS Istituto delle Scienze Neurologiche di Bologna, Pediatric Neuroradiology, Bologna, Italy; 4 IRCCS Istituto delle Scienze Neurologiche di Bologna, Pediatric Neurosurgery, Bologna, Italy; 5 Department of Biomedical and Neuromotor Sciences, University of Bologna, Unit of Pathology at Bellaria Hospital, Bologna, Italy; 6 Department of Experimental, Diagnostic and Specialty Medicine, Molecular Diagnostic Unit, Azienda USL di Bologna, University of Bologna, Bologna, Italy

**Keywords:** Congenital glioblastoma, Preterm infant, Cerebral ultrasound, Magnetic resonance imaging, GFAP, Case report

## Abstract

**Background:**

Congenital brain tumors are extremely rare in the neonatal population, and often associated with a poor prognosis. The diagnostic suspicion is often aroused at antenatal scans or postnatally, if clinical signs and symptoms of increased intracranial pressure become evident. We present a case of definitely congenital glioblastoma multiforme incidentally diagnosed in a preterm infant, aiming to raise clinical awareness on this condition and to highlight the challenges of the related diagnostic work-up.

**Case presentation:**

This female infant was born at 31 weeks’ gestation after an uneventful pregnancy. No abnormalities were detected at antenatal ultrasound scans and genetic tests. Head circumference at birth was on the 25th centile. A routine brain ultrasound scan performed on day 1 revealed a large, inhomogeneous lesion in the right cerebral hemisphere, with contralateral midline shift, which was confirmed by brain magnetic resonance imaging (MRI). Eye fundus and routine blood exams, including platelets count, coagulation screening and C-reactive protein, were normal.

Given the high risk of complications, surgical biopsy of the lesion was temporarily hold and a daily sonographic follow-up was undertaken. Although head circumference growth was steady on the 25th centile, progressive changes of the lesion were detected by cranial ultrasound.

The repeat MRI scans showed a significant enlargement of the mass, with contralateral midline shift and signs of intralesional and intraventricular bleeding. In view of this worsening, surgical resection was performed. The histological examination of the lesion biopsy documented a GFAP+ highly cellular neoplasm, with no mutation on SMARCB1 gene. At the molecular analysis, mutations on IDH and H3F3A genes were absent, whereas MGMT promoter was unmethylated. The diagnosis was grade IV glioblastoma IDH wild-type.

**Conclusions:**

Congenital glioblastoma multiforme is an extremely rare but highly aggressive neoplasm. Since intralesional biopsy is not often feasible in affected neonates, knowledge of the associated clinical and neuroradiological features is particularly important, as they can also add useful information on the neoplasm behavior. Specimens from open surgical resection allow to perform a definite histological analysis and an extended molecular characterization, with relevant prognostic implications.

## Background

With an estimated global incidence of 3.6 to 4.1 per 100.000 births, congenital brain tumors (CBTs) are extremely rare, accounting for only 0.5–1.9% of overall brain neoplasms in the pediatric population [1]. Based on the infant’s age at symptom onset, CBTs have been classified into definitely, probably and possibly congenital lesions, whose definition has varied over the past decades [2]. According to the latest modification [3], a symptom onset within the first 6 weeks of life defines a definitely congenital brain tumor.

Congenital CBTs can be incidentally detected at routine fetal scans as an intracranial mass, with or without hydrocephalus, whereas progressive macrocephaly is the postnatal sign most frequently observed [4]. The estimated overall survival rate lies around 30%, regardless of histology; however, the related clinical outcomes vary significantly in relation not only to the tumor’s size, location, histologic type and surgical resectability, but also to the infants’ condition at the time of diagnosis [4, 5].

We describe a case of definitely congenital glioblastoma multiforme, incidentally diagnosed in a preterm infant soon after birth, aiming to raise clinical awareness on the neuroimaging and pathological features associated with this condition and to highlight the challenges encountered in the related diagnostic work-up.

## Case report

**Fig. 1:**
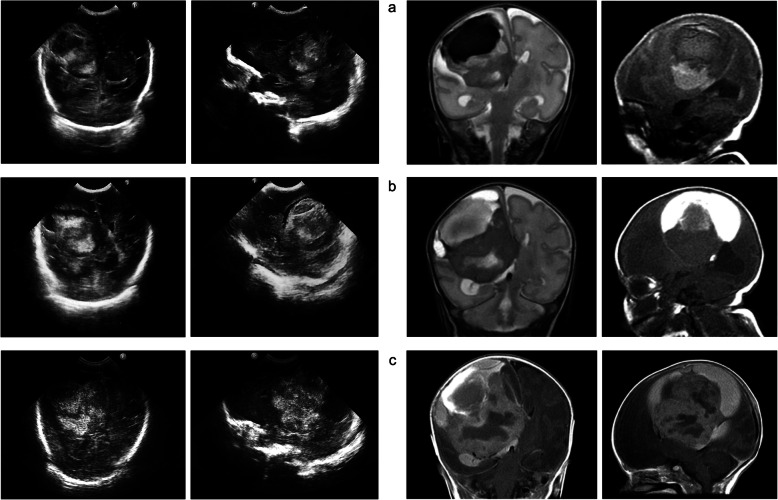
Cranial ultrasound (left columns) and magnetic resonance imaging (right columns) on day 1 (**a**), 18 (**b**) and 28 (**c**)

This Caucasian female infant was born at 31 weeks’ gestational age (GA) by spontaneous vaginal delivery in a dichorionic twin pregnancy. Birth weight was 1313 g (25th pc) and head circumference (HC) was 28 cm (25th pc). While recurrent miscarriages were noted at maternal history, pregnancy history was uneventful. Antenatal ultrasound scans showed no abnormalities (last scan performed at 28 weeks’ GA) and chorionic villus sampling documented a normal female karyotype. Due to her prematurity, the baby was admitted to the local Neonatal Intensive Care Unit shortly after birth. Clinical examination at admission was unremarkable; the anterior fontanel was soft and tender. On day 1, a routine cranial ultrasound scan (CrUSS) was performed, revealing a large inhomogeneous lesion in the right cerebral hemisphere, with contralateral midline shift (Fig. 1a, left).

A brain magnetic resonance imaging (MRI) scan without contrast medium was urgently performed on day 2, documenting a well-defined intra-axial right frontoparietal lesion, with evidence of intralesional hemorrhagic areas at different stages, homolateral ventricular bleeding and midline displacement due to mass effect (Fig. 1a, right). Additional diagnostic investigations, such as eye fundus and routine blood exams including platelets count, coagulation screening and C-reactive protein, were performed, turning out normal.

A joint consultation between the local neonatal, neurological and neurosurgical teams was performed, followed by an off-site neuroradiological and neurosurgical consultation; given the high risk of complications related to the infant’s GA and very low weight, surgical biopsy was temporarily hold. A MRI-angiography (MRA) was thus scheduled, and a daily sonographic follow-up of the lesion was undertaken.

Although the HC growth was steady on the 25th centile, progressive changes in the distribution of intralesional hypo- and hyperechoic areas and a slight increase in the size of the lesion were documented at CrUSS (Fig. 1b, left). MRA, performed on day 18, confirmed the increased lesion size and the evolutive changes of the intralesional bleeding; these findings were highly suggestive of an intra-axial neoplasm (Fig. 1b, right).

On this basis, a new neurosurgical consult was performed; since surgical risks were still remarkably high, clinical and sonographic follow-up was temporarily continued. The baby remained stable, with no signs of cranial hypertension nor focal neurologic symptom until day 28, when a remarkable enlargement of the lesion was observed at CrUSS (Fig. 1c, left); hence, an emergency MRI with contrast medium was performed. Compared to previous MRI scans, a further expansion of the polylobate mass, with recent signs of right intraventricular bleeding, was noted; the right ventricular dilatation and the contralateral midline dislocation were significantly increased, with concomitant subfalcine herniation. Evidence of peripheral enhancement was documented after intravenous contrast injection, whereas the central intralesional areas showed a prevalence of necrotic-cystic foci (Fig. 1c, right). In view of this worsening, the infant underwent surgical resection on day 30, at a weight of 1500 g. A significant intralesional bleeding occurred during the intervention. The intraoperative findings consisted of a highly vascular expansive mass, whose anatomical features allowed only a partial excision.

**Fig. 2:**
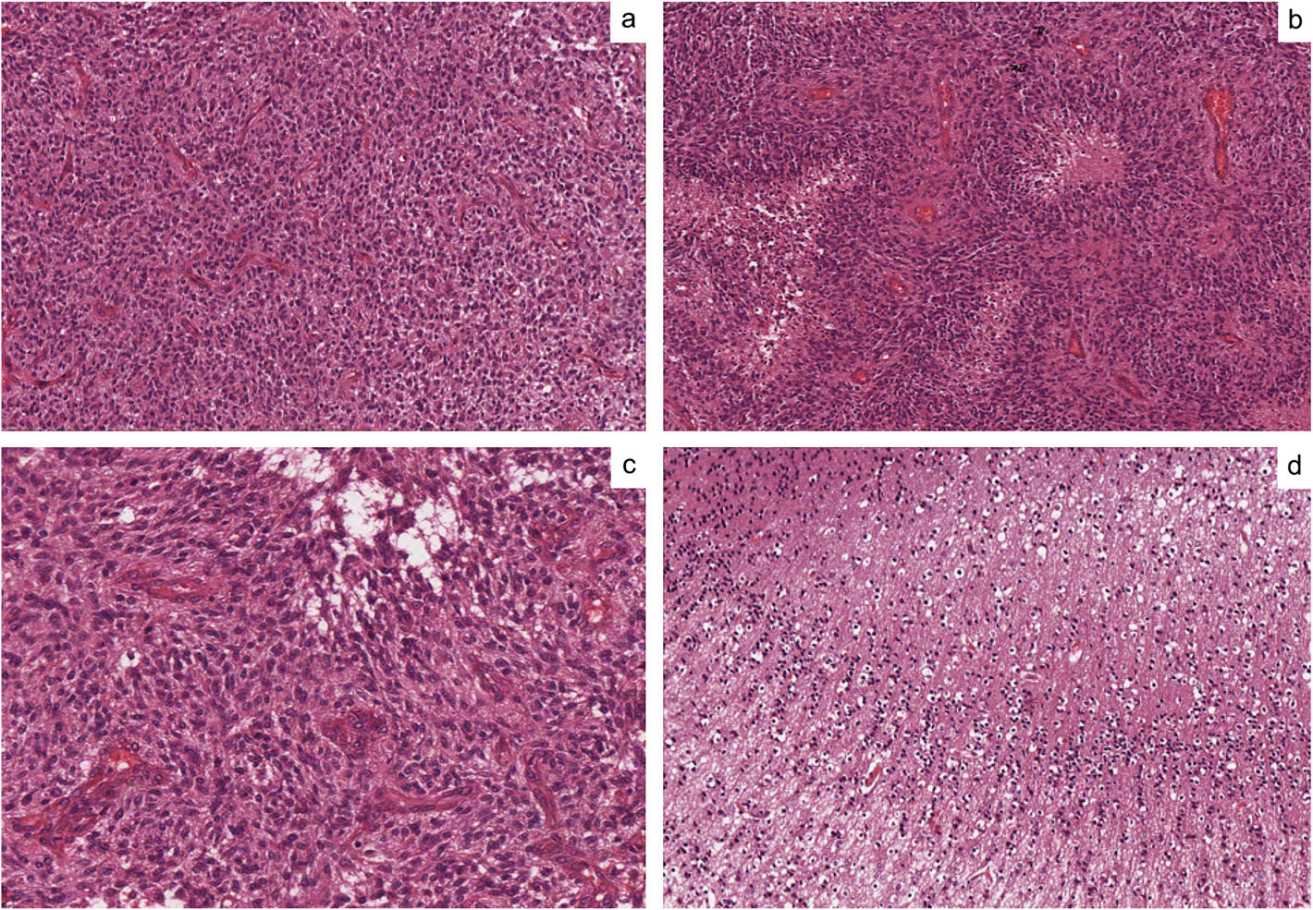
Histological features of the tumor biopsy: high cellularity with poorly differentiated glial cells with nuclear hyperchromasia, atypia and brisk mitotic activity (**a**); foci of palisading necrosis (**b**); high vascularization, characterized by hypertrophic endothelial cells or glomeruloid vessels (**c**); cortex infiltration by tumoral cells (**d**)

The histological examination of the lesion biopsy showed a highly cellular neoplasm, composed of poorly differentiated glial cells with nuclear hyperchromasia, atypia and brisk mitotic activity (Fig. 2a). Foci of palisading necrosis (Fig. 2b) and microvascular proliferation (hypertrophic endothelial cells or glomeruloid vessels) (Fig. 2c) were also noticed. The surrounding cortex was infiltrated by tumoral cells (Fig. 2d). Neoplastic elements expressed the glial marker GFAP (Glial Fibrillary Acidic Protein) and were negative for Synaptophysin and Neurofilament. In order to exclude an atypical teratoid rhabdoid tumor, antibody for INI-1 was performed and showed nuclear retention (no mutation on SMARCB1 gene). The growth fraction, determined by Ki-67 index, was 25%.

The molecular analysis was performed according to standard procedures with a GeneStudio S5 (ThermoFisher) NGS platform, targeting the relevant mutational hotspots of IDH1 (Exon 4), IDH2 (Exon 4) and H3F3A (Exon 1). MGMT promoter analysis was carried out according to previously reported protocols [6]. Mutations on IDH and H3F3A genes were absent, whereas MGMT promoter was unmethylated. The diagnosis was glioblastoma IDH wild-type, grade IV according to the WHO Classification.

An oncological consultation was performed; in light of the highly aggressive features of the neoplasm and the ensuing poor prognosis, together with the infant’s prematurity and low birth weight, palliative care was undertaken in conjunction with the family of the baby, who died 17 months later.

## Discussion

Glioblastoma multiforme (GBM) is a very rare but highly aggressive brain tumor, associated with a poor prognosis in both children and adults. Over the last 100 years, a total of 67 cases [7] of congenital GBM, characterized by symptom onset within the first year of life, have been reported. However, to the best of our knowledge, this is the first report of a definitely congenital GBM in a preterm infant.

In most of the cases previously reported, the diagnostic suspicion of congenital GBM was either aroused at antenatal scans and confirmed by fetal MRI [8], or raised postnatally by such clinical signs as abnormal HC, bulging fontanel, symptoms of raised intracranial pressure (e.g., feeding difficulties, vomit, apneas, lethargy, seizures etc.) [4, 7, 9]. In the present case, however, antenatal scans were unremarkable until no later than 3 weeks before birth; moreover, the tumor was diagnosed incidentally before becoming symptomatic, as no signs nor symptoms of intracranial hypertension were developed up to 4 weeks of life, when a major intralesional bleeding occurred.

Being non-invasive and easily accessible, CrUSS represents the first-line investigation in the diagnostic work-up of CBTs, which appear in the differential diagnosis of unexplained parenchymal hemorrhage, intraventricular hemorrhage, or brain abscesses [10]. Typical sonographic findings of congenital GBM consist of a unilateral heterogeneous mass, characterized by hyperechoic areas and cysts, most frequently located in the supratentorial region (92.2% of the cases reported) [7]. The mass typically occupies most of one hemisphere, and is often accompanied by contralateral midline shift and obstructive hydrocephalus [4]. Intralesional hemorrhage may be responsible for a rapid tumor growth and, as such, is not rarely reported as the initial imaging finding [4]. A serial CrUSS follow-up provides useful information on the neoplasm behavior (e.g., rapid growth over a short time period) and its possible response to treatment (e.g., development necrotic areas after chemotherapy, mass reoccurrence after resection) [10].

Multimodal MRI is an essential milestone for the differential diagnosis of CBTs. At MRI, GBM appears as a highly vascular lesion with a cystic-solid consistency, heterogeneous signal intensity and contrast enhancement, and a restricted diffusion due to its high cellularity and mitotic activity [11]. Although non-specific, evidence of an increased choline/creatine ratios and of a decrease in N-acetyl aspartate at MRI spectroscopy is further supportive of a glial origin [11].

Despite neuroimaging findings may be highly suggestive of GBMs, lesion biopsy allows not only a definitive diagnosis of GBMs, which is based on the evidence of such histological findings as high mitotic activity, microvascular proliferation and pseudopalisading, but also an extended molecular characterization [12]. However, intralesional biopsy may be burdened by high rates of complications [13], and does not change significantly the therapeutic management of GBM [14]. Hence, if an open surgical resection of the tumor is feasible, resection specimens represent a valid alternative to perform histological and molecular analysis.

In the present case, IDH and H3F3A mutations were absent and MGMT promoter was unmethylated. This is consistent with Gielen et al., who reported significantly lower rates of genetic alterations in congenital and infant GBM cases compared with older children and adults [15], hypothesizing that infant high-grade gliomas may represent a distinct genetic entity, with different pathogenesis and biological behavior, as it may also be suggested by reports of better clinical outcomes in this population [9, 16]. Nevertheless, while the optimal treatment for congenital GBM remains controversial and depends not only on the tumor’s but also on the infants’ characteristics, its prognosis is remarkably poor compared to other CBTs [1]. In this regard, following this diagnosis, it is particularly important to provide accurate prognostic information, and to involve the infants’ families in the decision-making process for the therapeutic or palliative management.

## Conclusions

Congenital glioblastoma multiforme is an extremely rare but highly aggressive neoplasm. Knowledge of the associated clinical and neuroradiological features is particularly important, as they can also add useful information on the neoplasm behavior. Specimens from open surgical resection allow to perform a definite histological analysis and an extended molecular characterization, with relevant prognostic implications for the therapeutic or palliative management.

## Data Availability

Data sharing not applicable to this article as no datasets were generated or analyzed during the current study.

## Abbreviations

CBTs: congenital brain tumors
CrUSS: cranial ultrasound scan
GA: gestational age
GBM: glioblastoma multiforme
GFAP: Glial Fibrillary Acidic Protein
HC: head circumference
MRA: magnetic resonance angiography
MRI: magnetic resonance imaging
